# Junctophilin 3 expresses in pancreatic beta cells and is required for glucose-stimulated insulin secretion

**DOI:** 10.1038/cddis.2016.179

**Published:** 2016-06-23

**Authors:** L Li, Z-F Pan, X Huang, B-W Wu, T Li, M-X Kang, R-S Ge, X-Y Hu, Y-H Zhang, L-J Ge, D-Y Zhu, Y-L Wu, Y-J Lou

**Affiliations:** 1 Insititute of Pharmacology and Toxicology, College of Pharmaceutical Sciences, Zhejiang University, Hangzhou 310058, China; 2 Key Innovation Team for Stem Cell Translational Medicine of Cardiovascular Disease of Zhejiang Province, College of Pharmaceutical Sciences, Zhejiang University, Hangzhou 310058, China; 3 Department of Pharmacy, The First Affiliated Hospital, College of Medicine, Zhejiang University, Hangzhou 310003, Zhejiang, China; 4 Cardiovascular Key Laboratory of Zhejiang Province, The 2nd Affiliated Hospital, College of Medicine, Zhejiang University, Hangzhou 310009, China; 5 Department of General Surgery, The 2nd Affiliated Hospital, College of Medicine, Zhejiang University, Hangzhou 310009, China; 6 The Population Council at the Rockefeller University, New York 10021, NY, USA; 7 Institute of Reproductive Biomedicine, the 2nd Affiliated Hospital, Wenzhou Medical University, Wenzhou 325027, China

## Abstract

It is well accepted that junctophilin (JPHs) isoforms act as a physical bridge linking plasma membrane and endoplasmic reticulum (ER) for channel crosstalk in excitable cells. Our purpose is to investigate whether JPHs are involved in the proper communication between Ca^2+^ influx and subsequent Ca^2+^ amplification in pancreatic beta cells, thereby participating in regulating insulin secretion. The expression of JPH isoforms was examined in human and mouse pancreatic tissues, and JPH3 expression was found in both the beta cells. In mice, knockdown of *Jph3* (si-*Jph3*) in islets decreased glucose-stimulated insulin secretion (GSIS) accompanied by mitochondrial function impairment. Si-*Jph3* lowered the insulin secretory response to Ca^2+^ signaling in the presence of glucose, and reduced [Ca^2+^]_c_ transient amplitude triggered by caffeine. Si-*Jph3* also attenuated mitofusin 2 expression, thereby disturbing the spatial organization of ER–mitochondria contact in islets. These results suggest that the regulation of GSIS by the K_ATP_ channel-independent pathways is partly impaired due to decrease of JPH3 expression in mouse islets. JPH3 also binds to type 2 ryanodine receptors (RyR2) in mouse and human pancreatic tissues, which might contribute to Ca^2+^ release amplification in GSIS. This study demonstrates some previously unrecognized findings in pancreatic tissues: (1) JPH3 expresses in mouse and human beta cells; (2) si-*Jph3* in mouse primary islets impairs GSIS *in vitro*; (3) impairment in GSIS in si-*Jph3* islets is due to changes in RyR2-[Ca^2+^]_c_ transient amplitude and ER-mitochondria contact.

Insulin secretion is associated with alterations of intracellular glucose metabolism, electrical excitability and Ca^2+^ handling of pancreatic beta cells.^1, 2, 3, 4, 5, 6^ In addition to association with Ca^2+^ release from type 2 ryanodine receptors (RyR2), insulin secretion from pancreatic beta cells is caused by glucose-stimulated [ATP] increase, Ca^2+^ entry via voltage-gated Ca^2+^ channels (VGCC) in plasma membrane (PM).^7, 8, 9, 10, 11, 12^ The resultant rise in intracellular Ca^2+^ concentration depends on the organization of VGCC and RyR2 within junctional membrane complexes (JMCs) in excitable cells,^13, 14^ but how the proper communication of Ca^2+^ influx and subsequent Ca^2+^ amplification influences the insulin secretion is not fully elucidated. Glucose-stimulated insulin secretion (GSIS) is the principal mechanism of insulin secretion. Loss or reduced GSIS are characteristic features of type 2 diabetes mellitus.^9^ The mechanism involved in triggering GSIS is well accepted as the K_ATP_ channel-dependent and -independent pathways,^6, 9^ the mitochondrial metabolism is the essential upstream core shared by both the pathways. However, to date, the link between Ca^2+^ amplification handling and ATP production during GSIS is not fully understood.

The family of junctophilin (JPHs) isoforms serves as a physical bridge and effectively contributes to the formation of JMCs for ion channel functional crosstalk in excitable cells.^15, 16^ Although pancreatic beta cells are electrically excitable,^6^ the role of JPHs in insulin release remains so far undefined. RyR2, inositol 1,4,5-trisphosphate receptor (IP3R) and sarco-endoplasmic reticulum Ca^2+^-ATPase 2b and 3 (SERCA2b, 3) in endoplasmic reticulum (ER) are closely associated with insulin release.^1, 3, 4, 17, 18^ Given that Ca^2+^ release amplification depends on the organization of VGCC and RyR2 within JMCs, whether the impairment of JMCs affects insulin secretion in beta cells should be urgently clarified. Knockout mice lacking the JPH isoforms display related pathological phenotypes,^15, 16, 19, 20, 21, 22^ indicating that JPHs are essential for the physiological communication. *In vivo* deletion of *RyR2* reduces Ca^2+^, ATP and oxidative metabolism, thereby leading to metabolic reprogramming and cell death.^23^ Furthermore, sleep deprivation upregulates *Jph3* transcription in mouse brain via stressful conditions through RyR-mediated intracellular calcium mobilization,^24^ suggesting that *Jph3* might also be a functional gene under stress in addition to its structural contribution. Given that Ca^2+^ signaling in GSIS includes the amplifying features through K_ATP_-independent pathway,^6, 9, 25, 26^ JPHs probably contribute to maintaining mitochondria function in beta cells. Importantly, the relationships between abnormal JPH isoforms and human diseases have also been confirmed. JPH2 deficiency resulted in hypertrophied and failing myocardium,^27, 28, 29, 30^ while *Jph3* mutation caused Huntington's disease-like-2.^31, 32^ Notably, strategies to maintain JPH2 level can prevent the progression from hypertrophy to heart failure,^33^ which highlights the potential therapeutic prospect of JPHs in beta cells in preventing the progress of type 2 diabetes. However, whether or not JPHs deficiency or mutation involves diabetic pathogenesis has so far not been proved.

The present study demonstrated that JPH3 is the major pancreatic isoform, which expressed in human and mouse pancreatic beta cells. In mouse islets, *Jph3* deficiency caused the acute damage of GSIS, which was associated with the impairment of ER–mitochondria axis integrity. Therefore, our finding reveals a novel functional role for JPH3 in maintaining GSIS under physiological and probably pathological conditions.

## Results

### Mouse and human pancreatic beta cells express JPH3

**Figure 1:**
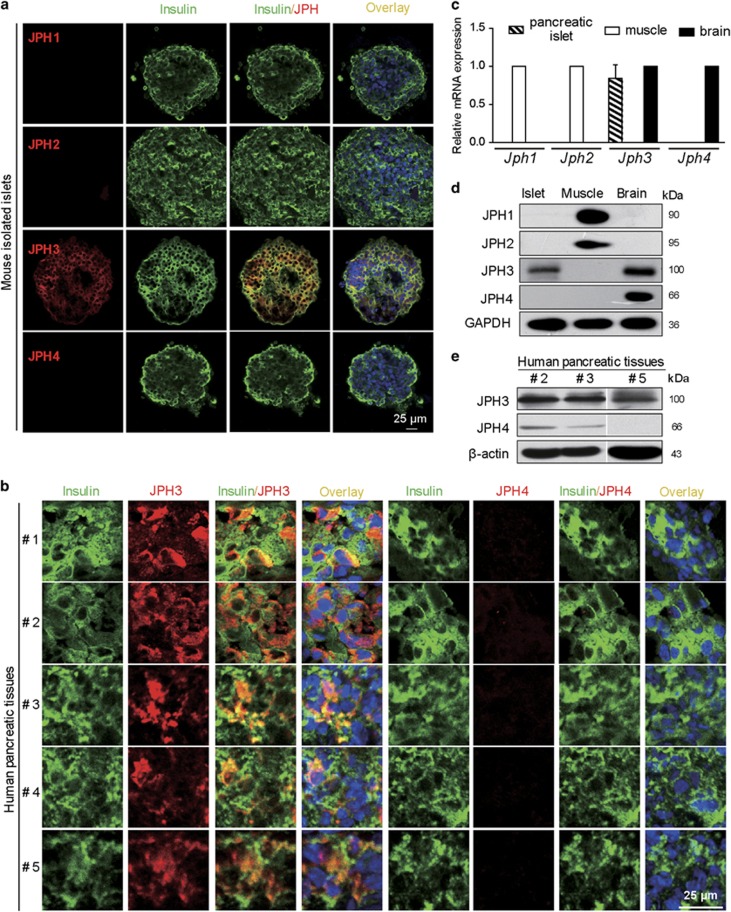
JPHs expression identification. (**a**) JPH3 in mouse islets. (**b**) JPH3 in human pancreatic tissues. (**c** and **d**) J*ph3* mRNA and JPH3 protein levels in mouse islets, mouse skeletal muscle and brain tissues as positive control. (**e**) JPH3 and JPH4 in human pancreatic tissues. Bar=25 *μ*m. See also Supplementary Table 1 for patient donor information

To explore the expression of JPH subtypes in pancreas, *in situ* immunofluorescence double staining of four JPH isoforms or JPH3 and JPH4 alone with insulin was performed in mouse primary islets or human pancreatic tissues. JPH3 selectively coexpressed with insulin in mouse and human pancreatic beta cells (Figures 1a and b). Quantitative RT-PCR (qRT-PCR) and Western blot assay further confirmed that only *Jph3* or JPH3 existed in mouse islets (Figures 1c and d) (also see Supplementary Figure 1). By contrast, human pancreas expressed not only JPH3 but also slight JPH4 in some donors (two out of three) (Figure 1e). Since pancreatic tissue contains vascular smooth muscle expressing JPH2, we did not check JPH1 and JPH2 expression.

### GSIS and mitochondrial condition in mouse si-*Jph3* islets

**Figure 2:**
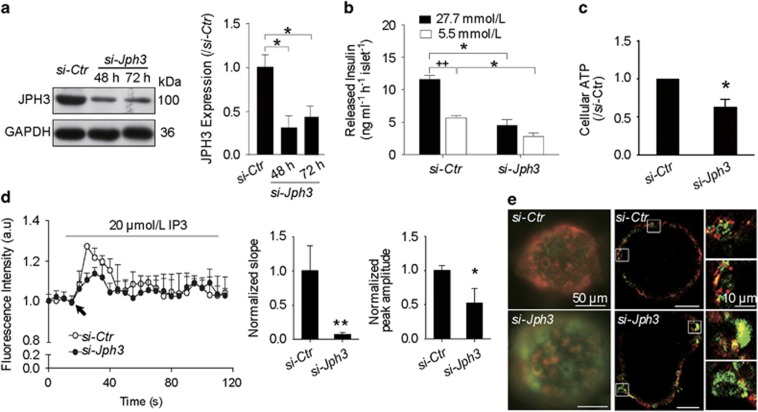
GSIS and mitochondrial function in si-*Jph3* islets. (**a**) *Jph3* silencing efficiency. (**b**) GSIS in response to high and low levels of glucose. (**c**) Intracellular ATP generation in the presence of glucose. (**d**) IP3-evoked [Ca^2+^]_m_ transients amplitude in permeabilized islets. Data are means±S.D. *n*=3 mice per group, with the minimum 30 islets for GSIS or 20 islets for ATP per mouse. *P<0.05, **P<0.01 *versus* si-*Ctr*. ^++^*P*<0.01 *versus* 27.7 mmol/L glucose. (**e**) Global ΔΨm in islets. Bar=10 *μ*m

To assess *Jph3* silencing efficiency, protein level of JPH3 was determined by Western blot. JPH3 was knocked down in the islets by 69.33±0.17% at 48 h and 57.35±0.09% at 72 h compared with control (Figure 2a). ELISA assay showed released insulin was 4.50±0.91 or 2.83±0.53 ng/ml/h per islet in si-*Jph3* islets compared with 11.61±0.63 or 5.68±0.36 ng/ml/h per islet in si-*Ctr* islets after incubated with 27.7 mmol/L (high) or 5.5 mmol/L (low) glucose, respectively (Figure 2b). This result indicated that acute attenuation of JPH3 dramatically impaired the GSIS. JPH3 was overexpressed in mouse islets by lentivirus (Supplementary Figure 2); however, GSIS was not affected in mouse Lenti-*Jph3* islets (Supplementary Figure 3), suggesting that extra JPH3 expression might be unnecessary for GSIS under physiological condition.

Meanwhile, we investigated the mitochondrial function in si-*Jph3* islets. Relative level of intracellular ATP induced by glucose was significantly affected in si-*Jph3* islets (Figure 2c). Given that IP3R-mediated Ca^2+^ flux was closely associated with mitochondrial Ca^2+^ concentration ([Ca^2+^]_m_) rise^34^ and ATP generation, we further examined the IP3-evoked mitochondrial Ca^2+^ transient amplitude in permeabilized si-*Jph3* islets. Representative fluorescence recordings showed that the initial slope and peak amplitude of [Ca^2+^]_m_ oscillations were considerably attenuated to 7.03±3.21 and 52.02±21.67% in si-*Jph3* islets compared with si-*Ctr* islets (Figure 2d). Considering that mitochondrial ATP synthesis is driven by ΔΨm,^5, 6, 10^ we further observed ΔΨm in si-*Jph3* islets. Using fluorescence microscope, the global islet demonstrated mitochondria depolarization with low ΔΨ (green) in si-*Jph3* islet as compared with active mitochondria with high ΔΨ (red) in si-*Ctr* islets (Figure 2e). Confocal laser fluorescence microscopy observation further exhibited several bigger bright-green sheets in si-*Jph3* islet compared with si-*Ctr* islet (Figure 2e), zoom, which was consistent with the reduced GSIS in islets.

### Ca^2+^-evoked GSIS and [Ca^2+^]_c_ transient amplitude in si-*Jph3* islets

**Figure 3:**
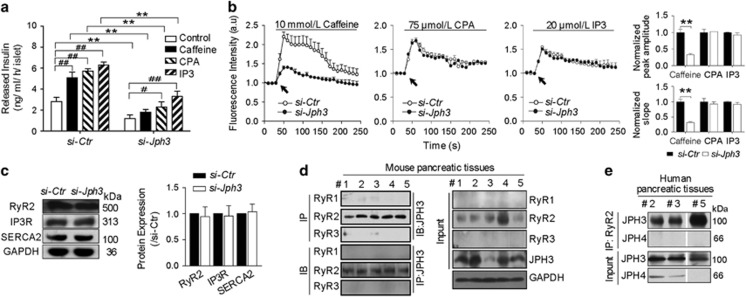
Insulin secretory response to Ca^2+^ signaling and [Ca^2+^]_c_ transient amplitude in si-*Jph3* islets. (**a**) Insulin secretory response to Ca^2+^ signaling in the presence of glucose, and (**b**) [Ca^2+^]_c_ transient amplitude in response to chemicals. *n*=3 mice per group, with the minimum 30 islets per mouse for insulin secretion. (**c**) Expression of Ca^2+^ releasing proteins of ER (*n*=3). (**d** and **e**) Co-immunoprecipitation showed JPH3 binds to RyR2 in mouse (*n*=5) and human (*n*=3) pancreatic tissues. Data are means±S.D. *P<0.05, **P< 0.01 *versus* si-*Ctr*. ^#^*P*<0.05, ^##^*P*<0.01 *versus* control in the same group

To explore whether the impairment in GSIS observed in si-*Jph3* islets is due to changes in intracellular Ca^2+^ levels, we measured Ca^2+^ signaling in response to glucose. All the chemicals-triggered insulin releases were significantly reduced in si-*Jph3* islets (Figure 3a). Interestingly, CPA and IP3 evoked-insulin releases displayed increase (2.29±0.50, 3.30±0.52 ng/ml/h per islet) in si-*Jph3* islets compared with 1.20±0.34 ng/ml/h per islet in the control (si-*Jph3*) islets. By contrast, caffeine lost triggering effect on insulin releases in si-*Jph3* islets (Figure 3a), suggesting a potential RyR2 Ca^2+^ signaling-dependent link between JPH3 expression and insulin secretion in islets. Chemicals-evoked [Ca^2+^]_c_ transient amplitude were further evaluated in si-*Jph3* islets. Quantification revealed that [Ca^2+^]_c_ transient amplitude was only blunted in response to caffeine in si-*Jph3* islets (Figure 3b), characterized by the decrease in both initial slope and peak amplitude of [Ca^2+^]_c_ oscillations to 31.90±3.20 and 32.80±3.29% compared with si-*Ctr* islets. The expression of Ca^2+^ channels or ATPase on ER was not affected in si-*Jph3* islets (Figure 3c). The finding that JPH3 or RyR2 can be selectively coimmunoprecipitated with the use of anti-RyR2 or anti-JPH3 antibodies suggests direct binding and regulation of RyR2 by JPH3 in mouse and human pancreatic tissue (Figures 3d and e). These data strongly suggested that *Jph3* knockdown in islets selectively affected the RyR2-Ca^2+^ signaling. The [Ca^2+^]_c_ transient amplitude and insulin secretion was not affected in Lenti-*Jph3* islets in response to the chemicals (Supplementary Figure 4), indicating that extra JPH3 expression is unnecessary in ER Ca^2+^ release under physiological condition.

### Si-*Jph3* disturbs ER–mitochondria contact and reduces Mfn2-related GSIS

The impairment of RyR2-Ca^2+^ signaling-related GSIS and mitochondrial function in si-*Jph3* islets prompted us to address whether JPH3 could participate in ER–mitochondria tethering. Using transmission electron microscopy, we confirmed that PM–ER junctional contact and ER–mitochondria contact were present in mouse beta cells (rod-like crystal-containing cell). Both contacts were well organized in a PM–ER–mitochondria or ER–mitochondria status in beta cell of mouse pancreatic tissue (Supplementary Figure 5). Unfortunately, the total size of si-*Jph3* islets harvested was too small to technically undergo the ultrastructural evaluation.

**Figure 4:**
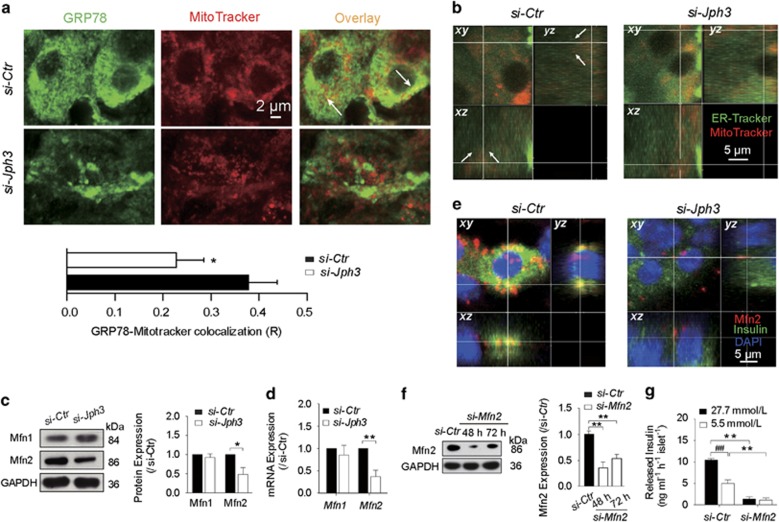
ER–mitochondria contact and Mfn2-dependent GSIS in si-*Jph3* islets. (**a**) The colocalization of ER and mitochondria in islets. (**b**) 3D confocal images of ER–mitochondria contact. (**c** and **d**) Mfn2 protein and *Mfn2* mRNA expression (*n*=3). (**e**) 3D confocal images of Mfn2 and insulin. (**f**) *Mfn2* silencing efficiency (*n*=3). (**g**) GSIS in si-*Mfn2* islets, *n*=3 mice per group, with the minimum 30 islets per mouse. Data are means±S.D. *P<0.05, ^**^P<0.01 *versus* si-*Ctr*. ^##^*P*<0.01 *versus* 27.7 mmol/L glucose

Considering the attenuated amplitude of [Ca^2+^]_m_ oscillation in si-*Jph3* islet, we next observed the spatial organization of ER–mitochondria contact in si-*Jph3* islets. The colocalization of ER (green) and mitochondria (red) fluorescent proteins display yellow area, which indicates that organelles are closer than ~270 nm.^35^ Confocal microscope observation demonstrated the colocalization areas of two organelles reduced by 43% in si-*Jph3* islets compared with si-*Ctr* islets (Figure 4a). The laser scanning confocal microscope through *xy*, *xz* and *yz* axes further showed that ER-Tracker (green) and MitoTracker (red) contact in a perpendicular position in the cell of si-*Ctr* islet. By contrast, ER and mitochondria separated from each other and their contact disappeared in a cell of si-*Jph3* islet (Figure 4b). All the data suggested that the spatial organized contact of ER and mitochondria was disrupted in si-*Jph3* islets.

We further focused on the major proteins, Mfn1 and Mfn2, tethering the organelles for Ca^2+^ shuttling through InsP3-gated channels from ER to mitochondria. Western blot analysis displayed that the expression level of Mfn2 was reduced to 47.84±18.47% in si-*Jph3* islets compared with si-*Ctr* islets, but the expression of Mfn1 was unchanged (Figure 4c). Real-time RT-PCR analysis showed that *Mfn2* expression level was also decreased to 54.37±5.53% in si-*Jph3* islets (Figure 3d). Notably, Mfn2 was enriched in cytoplasm and colocalized with insulin in si-*Ctr* islets, but fewer Mfn2 presented in si-*Jph3* islets (Figure 4e). To confirm whether the crucial effect of si-*Jph3* on GSIS was partly due to reduced Mfn2 expression, we observed the influence of *Mfn2* knockdown in GSIS of islets. Mfn2 was knocked down in the islets to 35.01±11.06% at 48 h (Figure 4f), and insulin release in response to high or low level of glucose was 1.38±0.49 or 1.12±0.54 ng/ml/h per islet in si-*Mfn2* islets compared with 10.53±0.27 or 5.03±0.78 ng/ml/h per islet in si-*Ctr* islets, respectively (Figure 4g). These results suggested that knockdown of *Jph3* resulted in the impairment of ER–mitochondria contact, which did associate with Mfn2 downregulation and contribute to GSIS dysfunction. The overexpression of JPH3 did not alter Mfn1 and Mfn2 expression in islets (Supplementary Figure 6), suggesting that basic JPH3 expression is enough to maintain the juxtaposition of ER and mitochondria in islets.

### Pgc-1*α* expression and localization in islets

**Figure 5:**
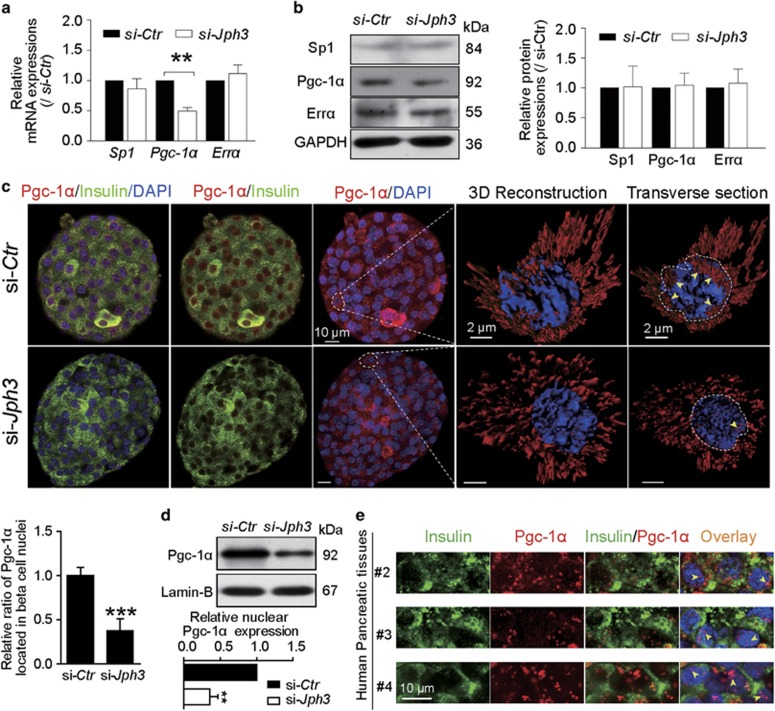
Pgc-1*α* in islets. (**a** and **b**) Expressions of *Pgc-1α* mRNA and Pgc-1*α* protein (*n*=3). (**c**) Confocal laser scanning exhibited the colocalization of Pgc-1*α* with insulin in si-*Ctr* and si-*Jph3* islets. Three-dimensional reconstruction and transverse section of a beta cell further displayed the localization of Pgc-1*α* within a nucleus (yellow arrowhead). Ratio of Pgc-1*α* existed in nuclei were showed in bar chart (*n*=4). (**d**) Expression of nuclear Pgc-1*α* in si-*Ctr* and si-*Jph3* islets (*n*=4). (**e**) Pgc-1*α* existed in nuclei of human beta cells. Data are means±S.D. *P<0.05, **P<0.01 *versus* si-*Ctr*

We next explored the possible signaling involved in the impairment of Mfn2 expression in si-*Jph3* islets. Sp1, Pgc-1*α* and Err*α* were believed to directly modulate Mfn2 expression in various tissues.^36, 37, 38^ Real-time RT-PCR assay showed that only *Pgc-1α* mRNA expression decreased to 49.25±5.98% in si-*Jph3* islets compared with si-*Ctr* islets (Figure 5a), implying that *Pgc-1α* transcription might partly regulate Mfn2 expression in islets. But Western blot analysis demonstrated that Pgc-1*α* did not significantly change in expression in si-*Jph3* islets (Figure 5b). Three-dimensional reconstruction by immunofluorescence image demonstrated that Pgc-1*α* distributed within nuclei in si-*Ctr* islets, but reduced in the nuclei of si-*Jph3* islets (Figure 5c). Relative ratio of Pgc-1*α* localized in DAPI was decreased to 37.75±13.24% in *si-Jph3* islets compared with *si-Ctr* islets (Figure 5c). Moreover, the relative nuclear Pgc-1*α* expression level declined to 35.75±10.95% in *si-Jph3* islets compared with *si-Ctr* islets as well (Figure 5d). The present results also indicated Pgc-1*α* nuclear translocation in the beta cells of mouse si-*Ctr* islet (Figure 5c) and existed in human pancreatic tissue with the possibility of nuclear translocation (without coexisting with insulin, Figure 5e). Pgc-1*α* coexpressed with PPAR*β*, a co-activator form, in nuclei of beta cells of human pancreatic tissue (Supplementary Figure 7), implying a possibility that RyR2-Ca^2+^ flux decrease could subsequently inhibit Pgc-1*α* activation in beta cells of si-*Jph3* islets as mentioned in muscle.^36^

### Ca^2+^ influx, ER stress or appoptosis-related protein expression in si-*Jph3* islets

**Figure 6:**
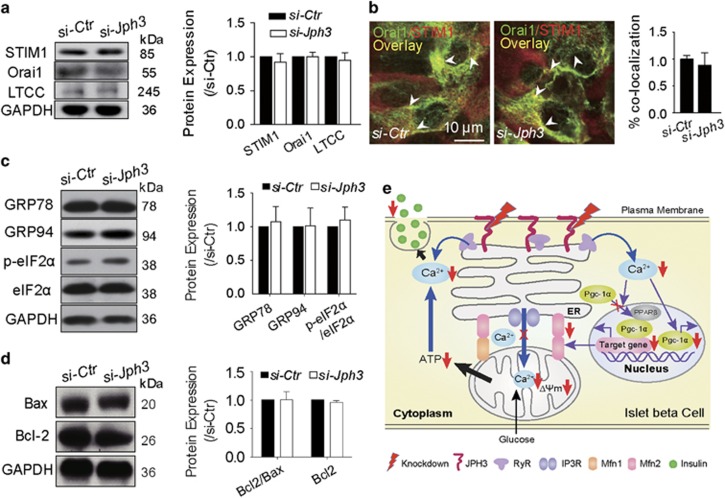
Ca^2+^ influx and ER stress or Bcl-2/Bax-related proteins in si-*Jph3* islets. Unchanged expression of Ca^2+^ influx-related proteins (**a**), colocalization areas (**b**), ER stress protein (**c**) Bcl-2/Bax ratio (**d**). *n*=3. (**e**) Schematic representation of the mechanism of JPH3-dependent GSIS in mouse beta cells

We next examined the effects of *Jph3* knockdown on protein expression linking to Ca^2+^ influx, including STIM1 in ER, and Orai1 or LTCC in PM in mouse islets. Western blot assay demonstrated that none of them were altered in expression in si-*Jph3* islets (Figure 6a) or Lenti-*Jph3* islets (Supplementary Figure 8). Immunofluorescence image indicated that the overlay of STIM1 and Orai1 in si-*Jph3* islets was similar to that in si-*Ctr* islets (Figure 6b), implying that the redistribution kinetics of the STIM1 binding to the Orai1 was unchanged in si-*Jph3* islets.

Given that ER stress could result from Mfn2 ablation^39^ or intracellular Ca^2+^ homeostasis disorder, we next tested whether attenuated Mfn2 expression and RyR2-Ca^2+^ flux would indirectly lead to ER stress in si-*Jph3* islets. Western blot assay showed that the early ER stress proteins GRP78 and GRP94, or p-eIF2*α* did not change in expression in si-*Jph3* islets compared with si-*Ctr* islets (Figure 6c), suggesting that knockdown of *Jph3* did not lead to ER stress in islets.

Anti-apoptotic Bcl-2 and pro-apoptotic protein Bax were located in the ER–mitochondria interface,^40^ and low ΔΨm could trigger apoptosis. Here, Western blot assay demonstrated that Bcl-2/Bax ratio was not changed in si-*Jph3* islets (Figure 6d). The schematic representation of the mechanism of JPH3-dependent GSIS in beta cells was described in Figure 6e.

## Discussion

The present study demonstrates that JPH3 is the major isoform in pancreas, which expresses in mouse and human pancreatic beta cells. We conditionally reduce JPH3 protein level using RNA interference in islet, thereby partly disrupting JMC structure in beta cells. We demonstrate that JPH3 is important for supporting the normal secretory function of mouse pancreatic beta cells *in vitro*. Si-*Jph3* in islets causes the impairment of mitochondrial function, decreases the insulin secretory response to Ca^2+^ signaling in the presence of glucose and reduces [Ca^2+^]_c_ transient amplitude in response to caffeine. Recently, a functional role for RyR2 in metabolism–secretion coupling both in mice and in humans is reported. Intracellular Ca^2+^ leak via RyR2 channels induces glucose intolerance associated with pancreatic beta cell ER stress, mitochondrial dysfunction and decreased insulin secretion.^1^ Our finding reveals that the interaction between JPH3 and RyR2 in human and mouse pancreatic tissues, which implicates JPH3 as a new regulator of RyR2 function, and downregulation of JPH3 might directly lead to RyR2 Ca^2+^ leak and impair RyR2-Ca^2+^ signaling. We speculate that the si-*Jph3* islets had defective K_ATP_-independent insulin secretion, which was associated with a reduction in RyR2-[Ca^2+^]_c_-related changes in GSIS and in glucose-stimulated increases in the ATP generation.

Impaired GSIS in islets is one of the characteristic features of type 2 diabetes mellitus.^9^ The effects of [Ca^2+^]_c_ on GSIS are still subject to debate. Some previous studies suggested that intracellular store (including RyRs) plays a minor role in GSIS.^18, 41^ Here, we show that acute knockdown of *Jph3* directly leads to impaired GSIS accompanied by RyR2 Ca^2+^ insufficiency, indicating that JPH3 is critical for maintaining RyR2-Ca^2+^ signaling in GSIS.

Our study further demonstrates that impaired GSIS is associated with damaged mitochondrial function in si-*Jph3* islets. Mitochondrial ATP synthesis is initiated by Ca^2+^ and driven by ΔΨm.^10^ In the presence of glucose, *Jph3* knockdown in islets affects IP3 evoked-Ca^2+^ rapidly shuttling through IP3R into the mitochondrial matrix, where Ca^2+^ activates Krebs cycle enzymes, leading to decreased ATP production. Therefore, JPH3 expression should be essential for triggering GSIS through K_ATP_-independent pathways. Our findings suggest that decrease of JPH3 is responsible for impaired GSIS, and also imply a causal link between JPH3 defects and type 2 diabetes.

The present results reveal that si-*Jph3* in islets also attenuated Mfn2 expression, thereby disturbing the spatial organization of ER–mitochondria contact, which probably also contributes to insufficient GSIS. The ER–mitochondria axis has been defined in muscle from obese or type 2 diabetic patients.^42^ Here, we further demonstrate that the spatial organized ultrastructural contacts of PM–ER–mitochondria do exist in mouse pancreatic beta cells, and that acute deficiency of JPH3 leads to alteration in ER–mitochondria contact morphology in islets. Moreover, IP3-evoked amplitude of [Ca^2+^]_m_ oscillations are considerably attenuated in permeabilized si-*Jph3* islets, which suggests that the organization of VGCC and RyR2 within JMCs are probably functionally associated with ER–mitochondria contact via RyR2-Ca^2+^ signaling. Notably, our findings showed that although human pancreatic tissue expresses JPH3 and a few JPH4, only JPH3 can directly bind to RyR2 in human pancreatic tissues, which is consistent with the binding and functional response of RyR2 by JPH3 in mouse si-*Ctr* islets. Homotypic and heterotypic interactions between Mfn2 on the ER and Mfn1 and 2 on mitochondria enable Ca^2+^ microdomains around open IP3R to propagate rapidly into the mitochondrial matrix, thereby increasing ATP production.^10, 43^ Our findings further reveal that although both Mfn1 and Mfn2 abundantly express in beta cells, *Jph3* knockdown in islets only selectively affects Mfn2 expression and *Mfn2* transcription. Furthermore, acute knockdown of *Mfn2* directly leads to impaired GSIS in islet, which highlights the possibility that decrease in Mfn2 expression due to JPH3 downregulation in beta cells might cause development of type 2 diabetes. Collectively, our findings, for the first time, suggest that the structural and functional ER–mitochondria axis integrity is of importance to maintain GSIS, and that *Jph3* knockdown significantly affects Mfn2 expression and subsequently influences the mitochondria function in beta cells.

Our study demonstrates that, among the three examined Mfn2 modulators, *Jph3* knockdown in islets selectively affects *Pgc-1α* mRNA expression at the transcription level. Previous researches demonstrated that the raise of intracellular Ca^2+^ concentration contributes to Pgc-1*α* nuclear translocation and transcription in myocytes and skeletal muscle.^36, 44^ PGC-1*α* takes part in shaping mitochondrial participation in calcium signalling that underlies its protective role against stress and proapoptotic stimuli in pathophysiological conditions.^45^ In addition, reduced RyR2 signaling can alter cardiac transcriptional programming, including *Pgc-1α* down-regulation, in a manner associated with cardiac pathology,^23^ and also cause abnormal Ca^2+^ handling in type 2 diabetic conditions.^17^ Our study further suggests that knockdown of *Jph3* might contribute to RyR2 Ca^2+^ signaling-related Mfn2 reduction via weakened Pgc-1*α* signaling in pancreatic beta cells. Taken together, JPH3 takes an important role in maintaining the suitable distance and crosstalk between ER and mitochondria via Pgc-1*α* pathway in beta cells. Recently, it is revealed that a Pgc-1*α*-PPAR*α*/*δ* partnership in nuclei can promote the target molecules transcription in skeletal muscle.^46^ PPAR*β* activation can also promote human and mouse embryonic stem cells differentiating into functional insulin-positive cells.^47^ Our findings imply that Pgc-1*α* and PPAR*β* in the nuclei of beta cells in human pancreatic tissue might act as co-activators, thereby contributing to regulating *Mfn2* expression. Therefore, it is valuable to further explore whether JPH3 takes a role in human GSIS in the future.

The present study showed that the protein expression and redistribution kinetics of the STIM1 binding to the Orai1 are unchanged in si-*Jph3* islets, indicating that *Jph3* knockdown does not affect store-operated Ca^2+^ entry function. In addition, si-*Jph3* caused Mfn2 attenuation or RyR2-Ca^2+^ insufficiency does not lead to ER stress and apoptosis-related protein expression, suggesting that GSIS insufficiency should not result from ER stress or apoptosis in si-*Jph3* islets.

In conclusion, JPH3 is the major pancreatic isoform expressed in both human and mouse pancreatic beta cells. Our findings suggested the physiological roles of JPH3 in beta cells and implicated JPH3 as an important candidate involved in the impairment of GSIS in type 2 diabetic conditions. Strategies to maintain JPH2 level can prevent the progression from hypertrophy to heart failure.^33^ Therefore, the dual functional roles for JPH3 in pancreatic beta cells, linking PM–ER and interaction with RyR2, may have implications for the development of new therapeutic strategies for type 2 diabetes.

## Materials and Methods

### Mouse pancreatic islet isolation

Eight-week-old male Balb/c mice were obtained from the Experimental Animal Center, Zhejiang University, China (Grade II, Certificate № SCXK 2008-0016). The experiments were conducted under the Guide for the care and use of laboratory animals (2011), and a protocol approved by the Institutional Animal Care and Use Committee of Zhejiang University (№ ZJU-2015-435-01), China. Islets were isolated by ductal collagenase V (Sigma Aldrich, St. Louis, MO, USA) injection from fully anesthetized mice^48^ and cultured in RPMI 1640 containing 20% FBS for further investigation.

### Human pancreatic tissue

Human pancreatic samples surrounding the cut tissues were obtained from surgical donors under prior informed consent procedure and with written consent from the Human Ethics Committee of the 2nd Affiliated Hospital (№ 2015-029), College of Medicine, Zhejiang University, China. Frozen 10 *μ*m consecutive sections were cut for immunocytochemistry analysis. Patient information could be found in Supplementary Table 1.

### Transfections with siRNA

Isolated islets were transfected with 100 nM siRNA against *Jph3*, *Mfn2* or negative control siRNA (*si-Ctr*) (Life Technologies, Carlsbad, CA, USA) using Lipofectamine RNAiMAX (Life Technologies) transfection agent according to the manufacturer's instructions. Sequences for siRNA were shown in Supplementary Table 2.

### Immunofluorescence image analysis

All immunostaining was conducted as previously described.^47^ Samples were probed with the primary antibodies as follows: Insulin, GRP78, STIM1 (Cell Signaling Technology, Danvers, MA, USA); JPH1, JPH2 (Thermo Fisher Scientific, Waltham, MA, USA); JPH3, JPH4, Mfn1, Mfn2, PPAR, Pgc-1*α* (Abcam, Cambridge, MA, USA); Orai1 (Pierce, Rockford, IL, USA). Then islets were incubated with secondary antibodies and DAPI (Sigma Aldrich). Images were acquired under Leica DMI3000B microscope (Leica Microsystems, Wetzlar, Germany) or Olympus FV1000 confocal microscope (CLSM, Olympus, Hertforshire, UK). Colocalization ratio of GRP78 and MitoTracker was calculated by Metamorph software (Molecular Devices, Sunnyvale, CA, USA) automatically. Relative ratio of Pgc-1*α* nuclear translocation was analyzed by Imaris software (Bitplane, Zurich, Switzerland).

### Western blot and coimmunoprecipitates analysis

Western blot was performed as previously described.^47^ Typically, sample lysates were resolved in SDS-PAGE and transferred onto PVDF membrane (Merck Millipore, Billerica, MA, USA). The PVDF membranes were incubated with the following primary antibodies (in addition to the above-mentioned): Err*α*, Sp1, IP3R, LTCC, Lamin B (Santa Cruz Biotechnology, CA, USA); RyR1, RyR2, RyR3 (Merck Millipore); SERCA2, GRP94, eIF2*α*, p-eIF2*α*, Bcl-2, Bax (Cell Signaling Technology); JPH1, JPH2, JPH3, JPH4, Mfn1, Mfn2, Pgc-1*α* (Abcam, Cambridge, MA, USA); GAPDH (Multisciences, Shanghai, China). Then samples were incubated with HRP-conjugated secondary antibodies (LK-GAM007, LK-GAR007, LK-RAG007, MULTISCIENCES). Blots were developed using enhanced chemiluminescence reagents (Pierce). Coimmunoprecipitates were analyzed by immunoblotting, using either anti-JPH3 or anti-RyR1, 2, 3 antibodies to detect the interaction between JPH3 and 3 RyR subtypes.

### RT-PCR analysis

Total RNA was isolated by Trizol (Life Technologies). For sqRT-PCR, reverse transcription was performed using M-MuLV first strand cDNA synthesis kit, and real-time PCR was performed using the PCR kit (Sangon, Shanghai, China). For qRT-PCR, RNA was treated by RT reagent kit (RR047A), and amplifications were performed using SYBR Premix Ex Taq Kit (TAKARA, Dalian, China). Primer sequences were displayed in Supplementary Tables 3 and 4.

### ELISA assay of GSIS

Islets were precultured in Krebs' Ringer Bicarbonate HEPES buffer containing 2.5 mmol/L glucose for 1 h. Cells were treated with different reagents for another hour. The supernatant was analyzed by Rat/Mouse Insulin ELISA Kit (#EZRMI-13 K, Millipore). The reagents were as follows: glucose 27.7 mmol/L or 5.5 mmol/L dissolved in KRBH buffer; 5 mmol/L caffeine (Zhejiang Institute for Food and Drug Control, Hangzhou, Zhejiang), 50 *μ*mol/L Cyclopiazonic acid (CPA, Tocris, Bristol, UK) and 10 *μ*mol/L D-myo-inositol-1,4,5-trisphosphate (IP3) (Tocris) dissolved in KRBH buffer containing 2.5 mmol/L glucose. The Krebs' Ringer Bicarbonate HEPES buffer consisted of 160 ml Buffer A, 200 ml Buffer B, 2.4 g HEPES, and 2 g BSA in a total volume of 1000 ml deionized water and filtered via 0.45 *μ*m and 0.22 *μ*m membranes. The Buffer A consisted of 34.6 g NaCl, 1.8 g KCl, 1.9 g CaCl_2_, 0.8 g KH_2_PO_4_, 1.5 g MgSO_4_•7H_2_O in 1000 ml deionized water. The Buffer B consisted of 13 g NaHCO_3_ in 1000 ml deionized water. Typically, for IP3 effect evaluation, islets were pretreated with 20 *μ*g/ml digitonin (Sigma Aldrich) for 30 min.

### Ca^2+^ transient measurement

Islets were loaded with X-rhod-1 AM (5 *μ*mol/L) and Rhod-2 (5 *μ*mol/L) (Life Technologies) for [Ca^2+^]_c_ and [Ca^2+^]_m_ (mitochondrial matrix Ca^2+^) indicator, respectively. Digitonin-permeabilized islets were used for IP3 experiment. Caffeine, CPA and IP3 were added to elicit Ca^2+^ release. Images were analyzed using software InVivo under live cell imaging system (IX S1, Olympus) by measuring fluorescence of individual islet at 549 nm and 576 nm, respectively. All analyses were processed as the relative fluorescence intensity. The slopes (from initiation to top) derived from the linear equation were calculated by Excel.

### Cellular ATP assay

Islets were incubated with 2.5 mmol/L glucose for 60 min.^49^ Then cells were analyzed by ATP bioluminescence assay kit (A095, Nanjing Jiancheng, China) following the manufacturer's instructions.

### Mitochondrial membrane potential (ΔΨm) measurement

For the determination of ΔΨm, cells were incubated with 2 *μ*g/ml JC-1 (5,5′,6,6′- tetrachloro-1,1′,3,3′-tetraethyl-benzimidazolylcarbocyanine iodide, T-4069, Sigma Aldrich) for 30 min at 37 °C in the dark. Cells were then washed by PBS and observed under Leica DMI3000B microscope. The red fluorescent J-aggregate indicates normal ΔΨm, while the green monomer fluorescence demonstrates low ΔΨm.

### Mitochondrion or ER probes staining

Islets were incubated with MitoTracker and ER-Tracker (Life Technologies) for 30 min following the manufacturer's instructions. Sections were photographed under Leica DMI3000B microscope (Leica Microsystems, Wetzlar, Germany) or Olympus FV1000 confocal microscope (CLSM, Olympus, Hertforshire, UK).

### Statistical analysis

Data were reported as means±S.D. Experiments were performed three times as repeats, and each time the islets were obtained from different mice. Comparisons of two groups used two-tailed student's *t*-test. Comparisons of multiple groups used ANOVA (GraphPad Prism 6; GraphPad software, San Diego, CA, USA). The differences were considered significant at *P*<0.05.

## Abbreviations

Bax: Bcl2-associated X protein
Bcl-2: B cell leukemia/lymphoma 2
[Ca^2+^]_c_: cytoplasmic matrix Ca^2+^
[Ca^2+^]_m_: mitochondrial matrix Ca^2+^
CPA: cyclopiazonic acid
eIF2*α*: eukaryotic initiation factor 2*α*
ER: endoplasmic reticulum
Err*α*: estrogen-related receptor *α*
GRP78: glucose-regulated protein 78
GRP94: glucose-regulated protein 94
GSIS: glucose-stimulated insulin secretion
IP3: D-myo-inositol 1,4,5-trisphosphate
IP3R: inositol 1,4,5-trisphosphate receptor
JMCs: junctional membrane complexes
JPH3: junctophilin 3
LTCC: L-type calcium channel
Mfn: mitofusin
Orai1: ORAI calcium release-activated calcium modulator 1
PGC-1*α*: peroxisome proliferator-activated receptor *γ* coactivator-1*α*
PM: plasma membrane
PPAR*β*: peroxisome proliferator-activated receptor *β*
RyR: ryanodine receptor
SERCA: sarco-endoplasmic reticulum Ca^2+^-ATPase
Sp1: specificity protein 1
STIM1: stromal interaction molecule 1
VGCC: voltage-gated Ca^2+^ channels
ΔΨm: mitochondrial membrane potential
