# Supplementary material for: Junctophilin 3 expresses in pancreatic beta cells and is required for glucose-stimulated insulin secretion
Source: Cell Death Dis. 2016 Jun 23;7(6):e2275–. doi: 10.1038/cddis.2016.179 (PMC5143404; doi:10.1038/cddis.2016.179)
Supplement: Supplementary Information [file cddis2016179x1.doc]

**Junctophilin 3 expresses in** **pancreatic beta cells and is required for glucose-stimulated insulin secretion**

Lu Li^1,2,3*^, Zong-fu Pan^1,2*^, Xin Huang^2,4^, Bo-wen Wu^1,2^, Tong Li^1,2^, Mu-xing Kang^5^, Ren-shan Ge^6,7^, Xin-yang Hu^2,4^, Yi-han Zhang^1,2^, Li-jun Ge^1,2^, Dan-yan Zhu^1,2^, Yu-lian Wu^5a^, Yi-jia Lou^1,2b^

^1^Institute of Pharmacology and Toxicology, ^2^Key Innovation Team for Stem Cell Translational Medicine of Cardiovascular Disease of Zhejiang Province, College of Pharmaceutical Sciences, Zhejiang University, Hangzhou 310058, China. ^3^Department of Pharmacy, The First Affiliated Hospital, College of Medicine, Zhejiang University, Hangzhou, Zhejiang 310003, China. ^4^Cardiovascular Key Laboratory of Zhejiang Province, ^5^Department of General Surgery, The 2nd Affiliated Hospital, College of Medicine, Zhejiang University, Hangzhou 310009, China. ^6^The Population Council at the Rockefeller University, New York, NY 10021, USA. ^7^Institute of Reproductive Biomedicine, the 2nd Affiliated Hospital, Wenzhou Medical University, Wenzhou 325027, China.

Supplementary information

This file includes:

Supplementary Methods

Supplementary Figures 1-7 and legends

Supplementary Table 4

**Supplementary Methods**

**Overexpression of JPH3**

Coding sequence of *Jph3* was ligated into lentiviral expression vector GV287 (Shanghai GeneChem, China). Lentiviral vector expressing eGFP alone was used as negative control. Islets were cultured in medium containing polybrene (Shanghai GeneChem, 5 μg/ml) and infected with lentivirus for 12 h.

**Cell viability assay**

Cell viability was determined by MTT (Amresco, LLC, MA, USA) assay, and measured according to the standard protocol.

**Immunofluorescence image analysis**

Samples were probed with the primary antibodies as follows: Insulin, PPAR β, Pgc-1α (Abcam, Cambridge, MA, USA). Then islets were incubated with secondary antibodies and DAPI (Sigma Aldrich). Images were acquired under Olympus FV1000 confocal microscope (CLSM, Olympus, Hertforshire, UK).

**Western blot analysis**

Typically, sample lysates were resolved in SDS-PAGE and transferred onto PVDF membrane (Merck Millipore, Billerica, MA, USA). The PVDF membranes were incubated with the following primary antibodies: IP3R, LTCC, RyR2 (Merck Millipore); SERCA2 (Cell Signaling technology); JPH3, JPH4, Mfn1, Mfn2 (abcam), GAPDH. Then samples were incubated with HRP-conjugated secondary antibodies (LK-GAM007, LK-GAR007, LK-RAG007, MULTISCIENCES). Blots were developed using enhanced chemiluminescence (ECL) reagents (Pierce).

**RT-PCR analysis**

For sqRT-PCR, reverse transcription was performed using M-MuLV first strand cDNA synthesis kit (B532435-0020) and real-time PCR was performed using the PCR kit (B532071-0100, Sangon, Shanghai, China). For qRT-PCR, RNA was treated by RT reagent kit (RR047A), and amplifications were performed using SYBR premix ex taq kit (638319, TAKARA, Dalian, China).

**ELISA assay of GSIS**

Islets were precultured in Krebs’ Ringer Bicarbonate HEPES buffer containing 2.5 mmol/L glucose for 1 h. Cells were treated with different reagents for another hour. The supernatant was analyzed by Rat/Mouse Insulin ELISA Kit (#EZRMI-13K, Millipore). The reagents were as follows: glucose 27.7 mmol/L or 5.5 mmol/L dissolved in KRBH buffer; 5 mmol/L caffeine (Zhejiang Institute For Food and Drug Control), 50 μmol/L Cyclopiazonic acid (CPA, Tocris, Bristol, UK) and 10 μmol/L D-myo-inositol-1,4,5-trisphosphate (IP3) (Tocris) dissolved in KRBH buffer containing 2.5 mmol/L glucose. The Krebs’ Ringer Bicarbonate HEPES buffer consisted of 160 ml Buffer A, 200 ml Buffer B, 2.4 g HEPES and 2 g BSA in a total volume of 1000 ml deionized water and filtered via 0.45 μm and 0.22 μm membranes. The Buffer A consisted of 34.6 g NaCl, 1.8 g KCl, 1.9 g CaCl_2_, 0.8 g KH_2_PO_4_, 1.5 g MgSO_4_·7H_2_O in 1000 ml deionized water. The Buffer B consisted of 13 g NaHCO_3_ in 1000 ml deionized water. Typically, for IP3 effect evaluation, islets were pretreated with 20 μg/ml digitonin (Sigma Aldrich) for 30 min.

**Ca^2+^ transient measurement**

The reagents were as follows: the RPMI 1640 containing 0.1% F-127 (1 g in 5 ml DMSO) was used to dissolve the X-rhod-1 AM and Rhod-2. After incubation with islets for 45 min at 37 °C and complete media treatment with islets for 15 min, replace media with wash buffer and measure fluorescence of individual islet at 549 nm and 576 nm, respectively. The wash buffer consisted of 2.816 g NaCl, 0.149 g KCl, 0.6506 g MgCl_2_, 0.965324 g HEPES, 0.015216 g EGTA, 0.0888 g CaCl_2_ in 1000 ml deionized water (pH 7.2).

**Transmission electron microscope**

Samples were dehydrated in acetones, permeated, and embedded in Polybed 812 epoxy resin. Ultrathin sections were cut and stained with 2% uranyl acetate, followed by 1% lead citrate. Sections were photographed by a transmission electron microscope Philips tecnai 10 (Royal Philips Electronics, Netherlands).

**Co-immunoprecipitation**

Western blot was used to detect JPHs or RyRs expression. Then samples were incubated with light chain specific secondary antibodies (Abbkine, Redlands, CA, USA).

**Statistical analysis**

Data were reported as means ± SD. Experiments were performed three times as repeats, and each time the islets were obtained from different mice. Comparisons of two groups used two-tailed student’s t-test. Comparisons of multiple groups used ANOVA (GraphPad Prism 6; GraphPad software, San Diego, CA, USA). The differences were considered significant at P<0.05.

**Supplementary Figures**

**
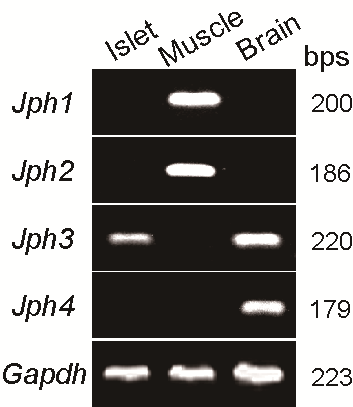
**

**Supplementary Figure 1.** *Jph3* mRNA in mouse islets, mouse skeletal muscle and brain tissues as positive control.

**
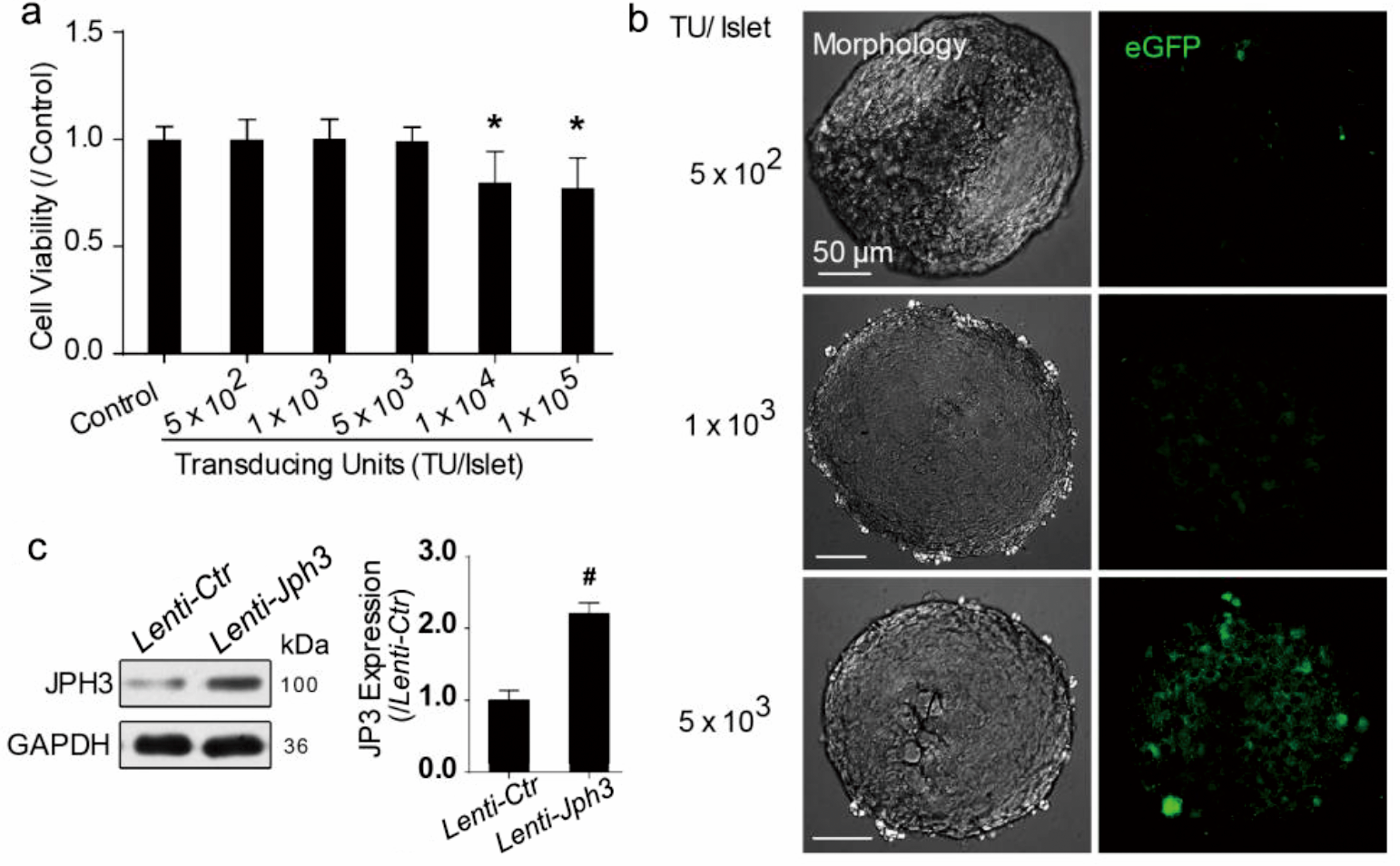
**

**Supplementary Figure 2. Overexpression of JPH3 in mouse islets.** **(a)** Cell viability determined by MTT assay after infected with lentivirus. **(b)** The green fluorescence of islets after infected with different transfecting units under confocal microscope, Bar=50 μm. **(c)** Protein expression of JPH3 after infected with 5× 10^3^ TU/Islet lentivirus for 5 d. Data are mean ± S.D., *n* = 3 mice per group, with the minimum 50 islets per mouse, **P* < 0.05 *vs.* Control, **^#^***P* < 0.05 *vs.* Lenti-*Ctr*.

**
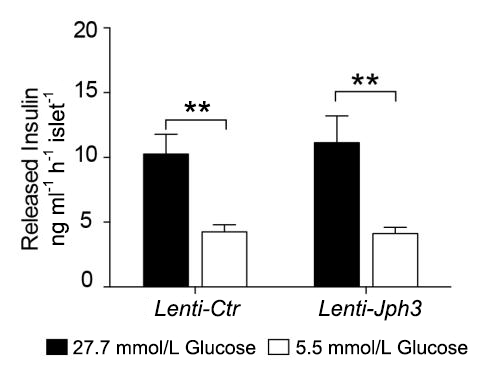
**

**Supplementary Figure 3. JPH3 overexpression did not alter the insulin release.** Insulin release after one hour treatment with 27.7 mmol/L, 5.5 mmol/L glucose in Lenti-*Ctr* or Lenti-*Jph3* infected islets. Data are means ± S.D., *n* = 3 mice per group, with the minimum 30 islets per mouse, ***P* < 0.01 *vs.* 27.7 mmol/L glucose of Lenti-*Ctr* or Lenti-*Jph3*, respectively.

**
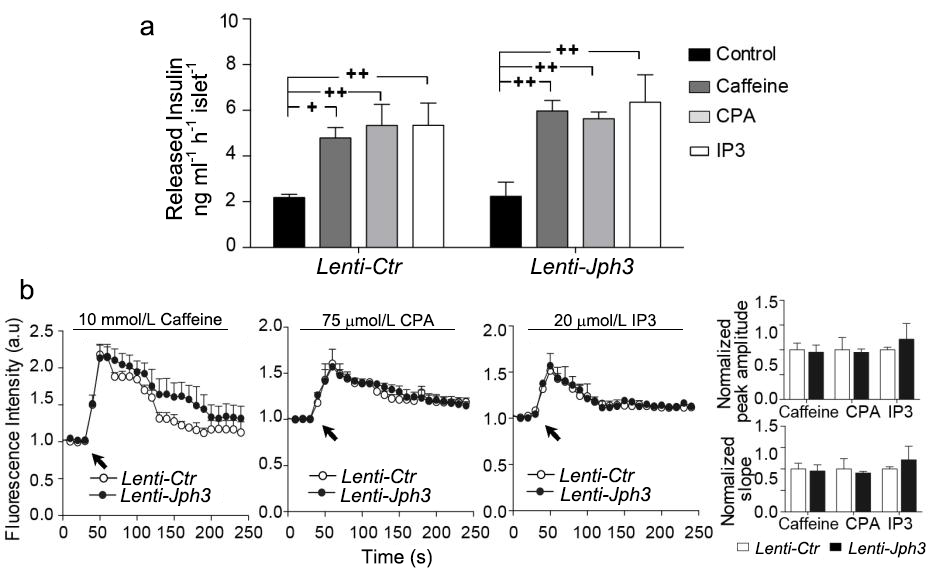
**

**Supplementary Figure 4. JPH3 overexpression did not alter the Insulin secretion and Ca^2+^ transients of islets**. **(a)** Insulin release after one hour treatment with 2.5 mmol/L glucose containing 5 mmol/L caffeine, 50 μmol/L CPA or 10 μmol/L IP3 in Lenti-*Ctr* or Lenti-*Jph3* infected islets. **(b)** A total of 10 mmol/L caffeine, 75 μmol/L CPA, and 20 μmol/L IP3 were added to elicit Ca^2+^ release, respectively. Individual islet [Ca^2+^]_C_ was recorded after loaded with x-Rhod-1 AM. The temporal resolution of the line scan was a profile of total fluorescence (arbitrary units). Data are means ± S.D., *n* = 3 mice per group, with the minimum 30 islets per mouse, ^+^*P*< 0.05, ^++^*P*< 0.01 *vs*. Control of Lenti-*Ctr* or Lenti-*Jph3*, respectively.


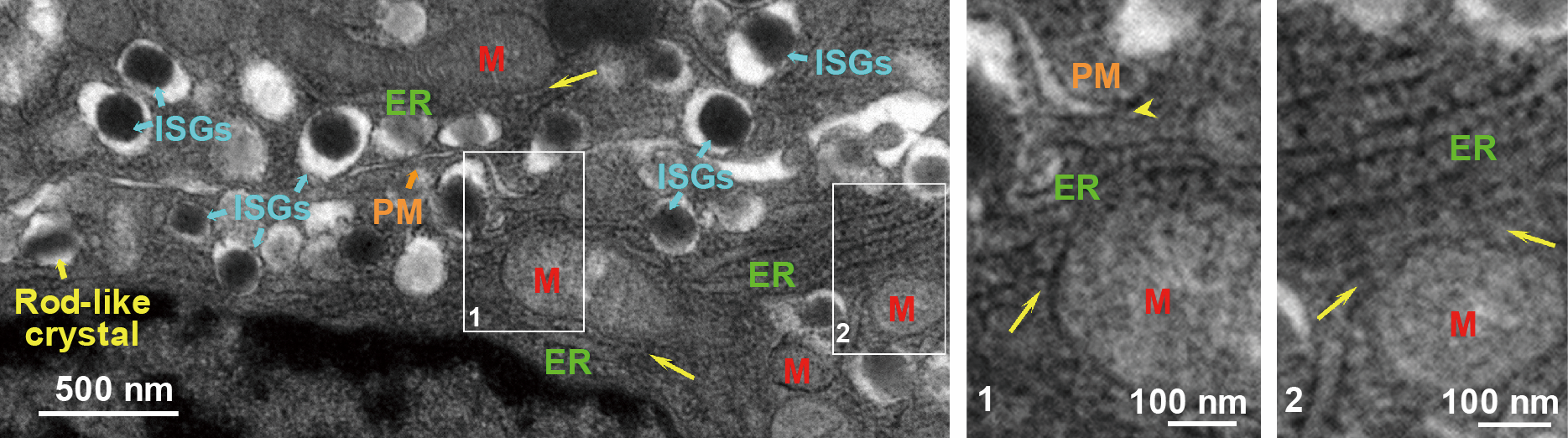


**Supplementary Figure 5. ER-mitochondria contact in mouse islet β cells.** The ultrastructures in mouse pancreatic tissue (M: mitochondria, ISGs: insulin secretory granules).

**
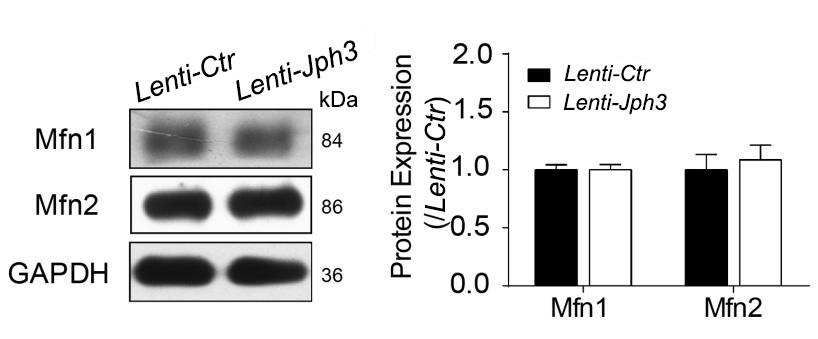
**

**Supplementary Figure 6. Protein expressions of Mfns did not change in islets after JPH3 overexpression**. Islets were infected with Lenti-*Ctr* or Lenti-*Jph3* and harvested at day 5. Data are means ± S.D., *n* = 3 mice per group, with the minimum 30 islets per mouse.

**
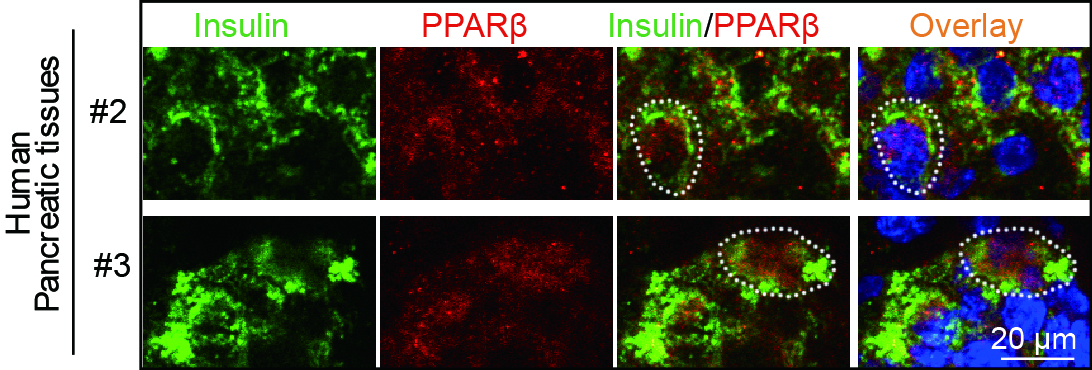
**

**Supplementary Figure 7. In human** **pancreatic tissue:** Immunofluorescence image indicated Pgc-1α nuclear translocation in beta cells and coexpression with PPARβ, a co-activator form, in nuclei of beta cells. Bar=20 μm.

**
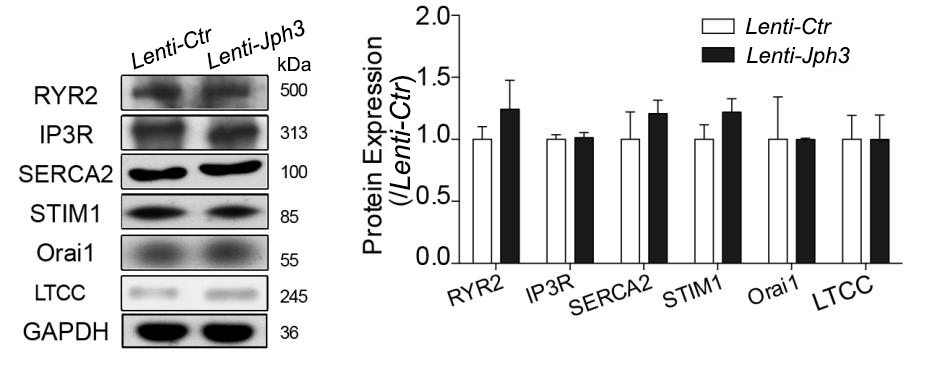
**

**Supplementary Figure 8. JPH3 overexpression did not alter the Ca^2+^ related protein expressions of islets**. Expression of Ca^2+^ channel related proteins in islets after infected with Lenti-*Ctr* or Lenti-*Jph3*. Data are means ± S.D., *n* = 3 mice per group, with the minimum 30 islets per mouse.

**Supplementary Tables**

**Supplementary Table 1 Basic information on the surgical donor patients** **under prior informed consent procedure and with written consent of the Human Ethics Committee**

| **Case ordinal (#)** | **Sex** | **Age** | **Human** **pancreatic samples from the cut tissues surrounding carcinoma** | **Case numbers** |
| --- | --- | --- | --- | --- |
| **1** | Female | 67 | Pancreatic carcinoma | 07438114 |
| **2** | Male | 50 | Duodenal carcinoma | 08838546 |
| **3** | Female | 65 | Bile duct carcinoma | 08896180 |
| **4** | Female | 60 | Intraductal papillary mucinous neoplasms | 00970222 |
| **5** | Male | 60 | Pancreatic carcinoma | 08933333 |

**Supplementary Table 2 Sequences for *siRNA***

| ***siRNA*** | **Forward (5’-3’)** | **Reverse (5’-3’)** |
| --- | --- | --- |
| Negative Control | UUCUCCGAACGUGUCACGUTT | ACGUGACACGUUCGGAGAATT |
| *Jph3* | GGUGAAUGGAAGAACGACATT | UGUCGUUCUUCCAUUCACCCA |
| *Mfn2* | UCCUCAAGGUUUAUAAGAATT | UUCUUAUAAACCUUGAGGACA |

**Supplementary Table 3 Sequences for the Primers and Conditions of sqRT-PCR**

| **Gene** | **Primer** | **Annealing temperature (°C)** | | **Cycle** |
| --- | --- | --- | --- | --- |
| *Jph1* | 5’-CCAACTCTGGCCCTAATTCC -3’  5’-ATTAAAGTGAGGGACAATGCCA -3’ | 58 | 35 | |
| *Jph2* | 5’-AACGACATGGGCTTGGCATA -3’  5’-GATAGGTTCCTCCGTCTGCG -3’ | 58 | 35 | |
| *Jph3* | 5’-CAGGGAAAACGGGCTTGAGT-3’  5’-CATCTTGGTCCTGGAGGCAG-3’ | 60 | 35 | |
| *Jph4* | 5’-TTCAGCCGAGGAAAAGGGAC-3’  5’-ATCCTGAGTGGCATGTGTGC-3’ | 60 | 35 | |
| *Gapdh* | 5’-ACGACCCCTTCATTGACCTCAACT-3’  5’-ATATTTCTCGTGGTTCACACCCAT-3’ | 60 | 28 | |

**Supplementary Table 4 Sequences for the Primers and Conditions of qRT-**PCR

| **Gene** | | **Primer** | | **Annealing temperature (°C)** | |
| --- | --- | --- | --- | --- | --- |
| *Jph1* | 5’-CCAACTCTGGCCCTAATTCC -3’  5’-ATTAAAGTGAGGGACAATGCCA -3’ | | 58 | |  |
| *Jph2* | 5’-AACGACATGGGCTTGGCATA -3’  5’-GATAGGTTCCTCCGTCTGCG -3’ | | 58 | |  |
| *Jph3* | 5’-CAGGGAAAACGGGCTTGAGT-3’  5’-CATCTTGGTCCTGGAGGCAG-3’ | | 60 | |  |
| *Jph4* | 5’-TTCAGCCGAGGAAAAGGGAC-3’  5’-ATCCTGAGTGGCATGTGTGC-3’ | | 60 | |  |
| *Mfn1* | | 5’-TCATTCTGAATAACCGTTGG-3’  5’-TCACTGCTGACTGCGAGA-3’ | | 60 | |
| *Mfn2* | | 5’-AAGTCCGGGAAGCTGAAAGT-3’  5’-TCTCGGTTATGGAACCAACC-3’ | | 60 | |
| *Pgc-1α* | | 5’-TGTTCCCGATCACCATATTCC-3’  5’-TGCTTCTGCCTCTCTCTCTGTTT-3’ | | 60 | |
| *Sp1* | | 5’GGCCTCCAGACCATTAACCTCA-3’  5’-TCATGTATCCCATCACCACCAGA-3’ | | 60 | |
| *Errα* | | 5’-GATGTGGCCTCTGGCTACCACTA-3’  5’-TCCGCTTGGTGATCTCACACTC-3’ | | 60 | |
| *Gapdh* | | 5’- TCCATGACAACTTTGGCATTG-3’  5’- CAGTCTTCTGGGTGGCAGTGA-3’ | | 60 | |
